# Acetylcholine receptor binding antibody–associated myasthenia gravis, myocarditis, and rhabdomyolysis induced by tislelizumab in a patient with colon cancer: A case report and literature review

**DOI:** 10.3389/fonc.2022.1053370

**Published:** 2022-12-08

**Authors:** Shengnan Wang, Danping Peng, Hao Zhu, Wanwan Min, Mengru Xue, Rui Wu, Yanqing Shao, Lin Pan, Mingqin Zhu

**Affiliations:** ^1^ Department of Neurology, The First Hospital of Jilin University, Changchun, China; ^2^ Department of Infectious Diseases, The First Hospital of Jilin University, Changchun, China; ^3^ Department of Hepatology, The First Hospital of Jilin University, Changchun, China; ^4^ Clinical College, Jilin University, Changchun, China

**Keywords:** tislelizumab, myocarditis, myositis, Myasthenia Gravis, immune-related adverse events, locally advanced colorectal cancer

## Abstract

Despite the intriguing therapeutic prospects offered by immune checkpoint inhibitors (ICIs), immune-related adverse events (irAEs) become an increasingly important safety issue. Herein, we report a patient with locally advanced colorectal cancer (LACRC) who received anti-programmed cell death protein 1 (PD-1) (tislelizumab) therapy, then developed weakness of the limbs and drooping eyelids. He experienced sequential irAEs including severe myasthenia gravis, myocarditis, and rhabdomyolysis. Although many irAEs caused by tislelizumab have been reported, the cooccurrence of severe myasthenia gravis, myocarditis, and rhabdomyolysis caused by tislelizumab has not been described. The patient responded well to methylprednisolone and intravenous immunoglobulin therapy. This case illustrates the severe toxicity caused by ICIs, highlighting the importance of early prevention, early diagnosis, and appropriate management of irAEs. Multidisciplinary discussions should be held to improve the prognosis of patients.

## Introduction

In recent years, immune checkpoint inhibitors (ICIs) have been used to treat multiple types of tumors. Programmed death-1 (PD-1) and programmed death-ligand 1 (PD-L1) immuno-checkpoint inhibitors represent one of the most significant breakthrough in the treatment of advanced malignancies (1). PD-1 inhibitors facilitate restore the endogenous anti-tumor T cell response by blocking the binding of PD-1 to PD-L1 and PD-L2. PD-1 inhibitors, such as nivolumab, have been approved by the US Food and Drug Administration (FDA) for a wide range of cancers, such as melanoma, renal cell carcinoma (RCC), colon cancer, bladder cancer, and Hodgkin’s lymphoma since 2014 (2). Despite the impressive efficacy, ICI-related toxicities (i.e., immune-related adverse events and irAEs) should not be neglected, as many irAEs such as myocarditis and rhabdomyolysis are covert and fatal (3, 4). Therefore, the use of ICIs should be accompanied by vigilance against the occurrence of serious irAEs. Herein, we report a patient with locally advanced colorectal cancer (LACRC) who developed tislelizumab-induced multiple organ irAEs including myasthenia gravis, myocarditis, myositis, liver damage, and kidney damage. The patient was admitted to the neurological care unit (NCU) and his symptoms improved significantly after intravenous immunoglobulins (IVIGs) and corticosteroids treatments. We hope to provide reference for the prevention and treatment of clinically related adverse reactions.

## Case report

**Figure 1:**
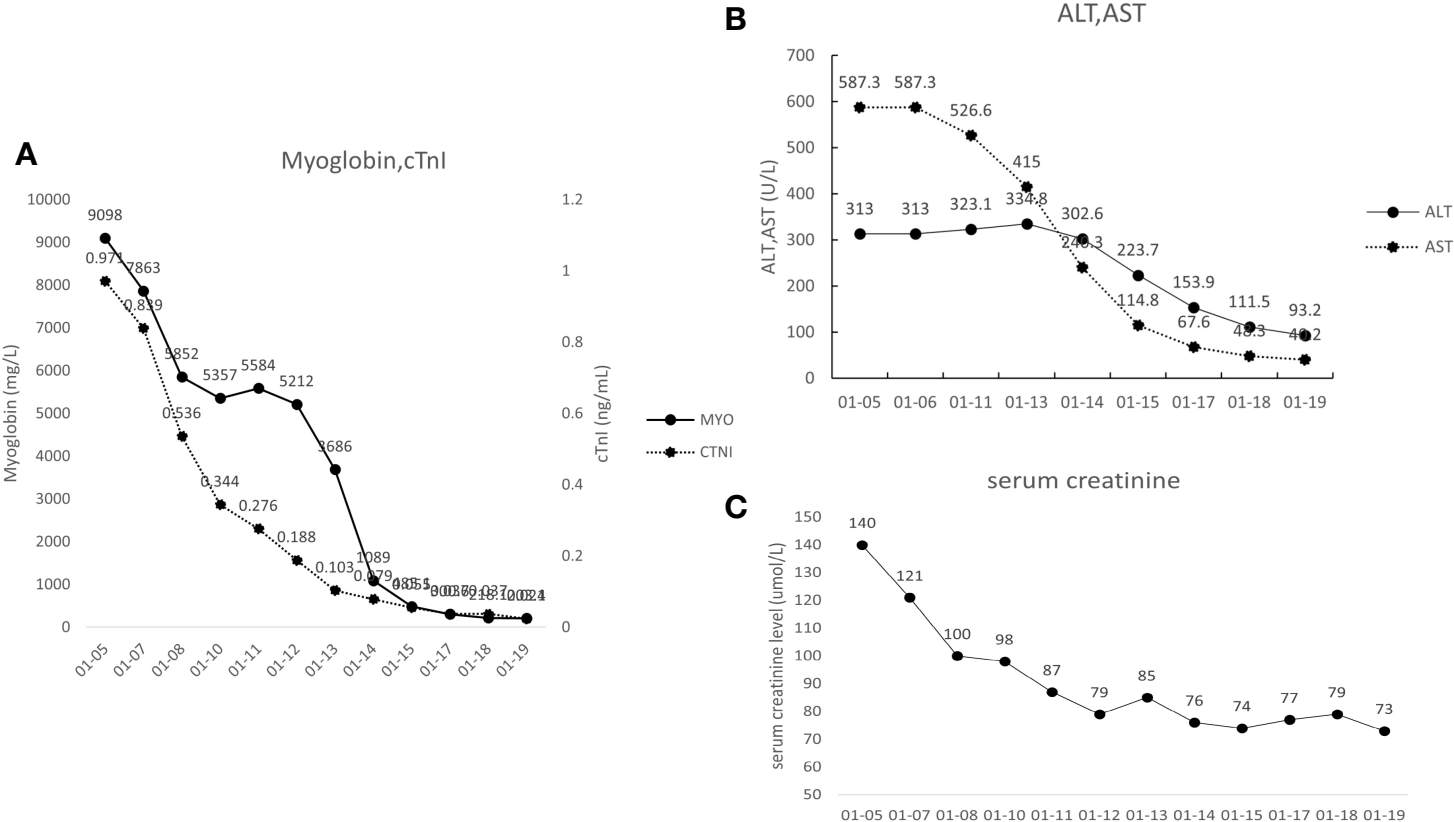
**(A)** Changes to the patient’s cardiac markers. Myo, myoglobin; cTNT, cardiac troponin T. **(B)** Changes to the patient’s liver function indexes. ALT, alanine aminotransferase; AST, aspartate aminotransferase. **(C)** Changes to the patient’s serum creatinine.

**Figure 2:**
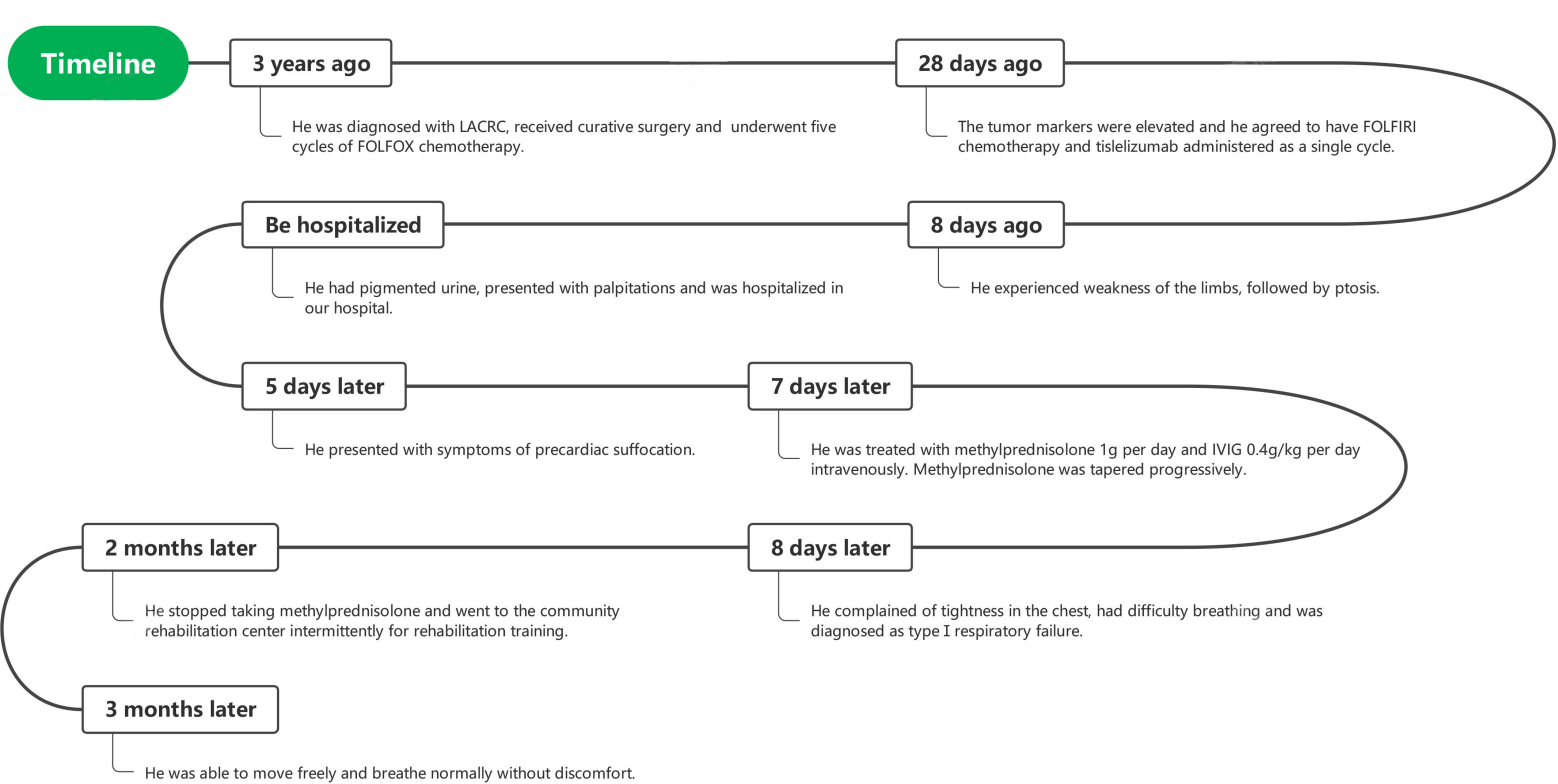
Flow chart of patient condition during simulation progression.

A 65-year-old man was diagnosed with LACRC and received curative surgery in 3 years ago due to repeated, intermittent hematochezia and black stool. He was previously healthy with no autoimmune medical history. The tumor was staged as a T3N0Mx rectal adenocarcinoma as per the TNM (American Joint Committee on Cancer TNM Staging Handbook) staging system. He underwent five cycles of 5-fluorouracil and oxaliplatin (FOLFOX) chemotherapy, and his cancer was stable during the follow-up period from 2018 to 2021. A year ago, his carcinoembryonic antigen (CEA) levels were elevated to 25.43 ng/ml (normal range 0–5 ng/ml) and enhanced CT of the abdomen revealed recurrence of colon cancer and multiple enlarged metastases in the right anterior superior diaphragm and left inguinal region, suggesting the progression of disease. Therefore, molecular genotyping and microsatellite status was performed, and he was diagnosed as high microsatellite instability/defective mismatch repair (MSI-H/dMMR) phenotype colorectal cancer. Because therapeutic failure appears with FOLFOX therapy, then FOLFIRI chemotherapy (folic acid/fluorouracil/irinotecan) was applied for the 6^th^ cycle chemotherapy without adverse effects. Tislelizumab was given to the patient intravenously (200 mg/day for 20 days, BeiGene, China) since December 9, 2021, and FOLFIRI scheme was conducted concomitantly. Twenty days later, the patient experienced weakness of the limbs, followed by bilateral eyelid ptosis, and was admitted to our department. Three days after admission, he had pigmented urine and developed cardiac symptoms such as palpitations. Neurological examination showed that the patient had head drop and complete bilateral gaze paralysis in all directions. Muscle strength of the limbs was 4/5, and the deep tendon reflex was absent. Electromyography (EMG) was normal. His neostigmine test result was positive, which showed clinically significant improvements in quantitative Myasthenia Gravis score (QMG) (> 3 points) (5). Laboratory results revealed significantly elevated levels of myocardial enzymes: creatine kinase (CK) (11920 U/L, normal range 50–310 U/L), CK isoenzyme (CK-MB) (244.2 U/L, normal range 0–25.0 U/L), lactate dehydrogenase (LDH) (1582 U/L, normal range 120 - 250 U/L), α-hydroxybutyric acid (AHB) (1120 U/L, normal range 72–182 U/L), myoglobin (7863 mg/L, normal range 1.5–70 mg/L) and cardiac troponin (cTnI) (0.839 ng/ml, normal range < 0.034 ng/ml). Abnormal liver function was also observed as evidenced by increased levels of alanine aminotransferase (ALT) (313.0 U/L, normal range 9–50 U/L) and AST (587.3 U/L, normal range 15 - 40 U/L). Additionally, his urine protein was positive, urine red blood cell count was 39.1, and his blood urea was 10.17 mmol/L (normal range 2.86–7.14 mmol/L); serum creatinine level was 140 umol/L (normal range 57–111 umol/L), suggesting kidney injury. We demonstrated reduced cortisol levels in the morning (75.89 nmol/L, normal range 244–619 nmol/L) and (77.88 nmol/L, normal range 244–619 nmol/L) at night. His ACTH (adrenocorticotropic hormone) levels were also decreased in the morning (0.22 pmol/L, normal range 1.6–13.9 nmol/L) and (0.22 pmol/L, normal range 1.6–13.9 nmol/L) at night. The patient had hypopituitarism as indicated by low plasma cortisol levels and plasma ACTH levels. His tumor marker CEA was elevated (9.47 ng/ml, normal range 0–5 ng/ml), however, was significantly decreased compared with levels prior to tislelizumab treatment. Antinuclear antibodies (ANA) were 1:100 positive. Serum IgG, IgA, IgM, and complement C3 and C4 were all within normal range. Other autoimmune tests, including antimitochondrial antibody (AMA), perinuclear anti-neutrophil cytoplasmic antibody (p-ANCA), cytoplasmic anti-neutrophil cytoplasmic antibody (c-ANCA), and ceruloplasmin, were all within normal range. Cerebrospinal fluid (CSF) routine test showed no abnormalities in cell counts, protein, and IgG levels. His autoimmune neuromuscular disease antibody tests by radioimmune assay (RIA) revealed elevated levels of anti-acetylcholine receptor antibody (AChR) (0.947 nmol/L, normal range 0–0.5 nmol/L), and anti-titin antibody was determined by enzyme-linked immunosorbent assay (ELISA) and the results showed positive (2.537, normal range < 1). The rest tests for autoimmune neuropathies or paraneoplastic syndrome were all negative. Grade 4 tislelizumab-induced irAEs including MG, myocarditis, myositis, liver damage, and kidney damage was diagnosed according to the Common Terminology Criteria for Adverse Events (CTCAEs) v5.0. The patient received methylprednisolone at 1g/day intravenously for 3 days, afterward the dose of methylprednisolone was gradually tapered down. IVIG treatment was given at 0.4g/kg per day for 3 days simultaneously. Despite no significant improvement of clinical symptoms, the patient’s myocardial enzymes decreased gradually 2 days after the treatment. Five days after admission, the patient complained tightness of the chest and had difficulty in breathing. Arterial blood gas analysis showed type I respiratory failure. The patient was transferred to the NCU for respiratory support, and dyspnea improved 6 days later. Non-invasive positive pressure ventilation was applied for respiratory support. Approximately 20 days after admission, the levels of CK-MB, CK, LDH, AHB, Myo, ALT, AST, troponin, urine microproteins, and kidney function gradually returned to normal (**Figure 1**). The patient was discharged from hospital 23 days after admission with conventional home ventilator support (intermittent), as he still had difficulty in breathing when he discharged from hospital. After discharge, the patients went to the community rehabilitation center intermittently for rehabilitation training, and the serum indicators (liver enzymes, CK, troponin, etc.) were closely monitored in the community hospital. The patient regained the autonomous walking and weaned off the breathing machine entirely 3 months later. (The symptoms and treatment of the patient were summarized in **Figure 2**).

## Discussion

We present a 65-year-old male patient with tislelizumab-induced multiple organ irAEs, who manifested as weakness of the limbs, drooping eyelids, oculomotor nerve paralysis, dyspnea, rhabdomyolysis, and palpitations. The patient responded well to corticosteroids and IVIG treatment; the symptoms gradually improved. This is the first report of occurrence of polymyositis with MG in colorectal cancer (CRC) patients treated with tislelizumab.

LACRC is defined as CRC stage II (cT3–4, N0)/stage III (any cT, N+) (6). Except for patients with oligometastatic disease, most patients with LACRC have incurable disease (7). The 5- and 10-year survival rates of patients after successful surgical or ablative interventions are approximately 40 and 20%, respectively (8–10). Traditional surgical resection cannot meet the needs of all patients, and MSI-H/dMMR LACRC has low sensitivity to chemotherapy. Most clinical studies demonstrated that patients with MSI-H/dMMR solid tumors received obvious benefits from ICIs (11–13). Thus, ICIs may be a breakthrough for the treatment of colon cancer.

PD-1 inhibitor tislelizumab (BGB-A317) is a humanized IgG4 monoclonal antibody that inhibits the binding of FcγR to macrophages, thus eliminating antibody-dependent phagocytosis and improving T cell clearance (14, 15). Chinese authorities approved tislelizumab for Hodgkin’s lymphoma by the National Medical Products Administration (NMPA) in December 2019. PD-L1/PD-L2–associated cell signaling can lead to cytokine production and a reduction in tumor cell death associated immune response. The PD-1/PD-L1 cell signaling can be blocked by tislelizumab (15). Tislelizumab was approved in China due to its antitumor potential in a variety of malignant tumors including relapse or refractory classical Hodgkin lymphoma, urothelial carcinoma, non-small cell lung cancer, and hepatocellular carcinoma. Moreover, results from clinical trials showed tislelizumab had beneficial effects in treating tumors in multiple systems. On March 11, 2022, the NMPA officially approved tislelizumab for adult patients with MSI-H type or dMMR type solid tumor, which brings new options and hope for the treatment of many advanced solid tumors including CRC.

However, due to the increase in clinical use of PD-1 inhibitors, the associated irAEs increased as well (16). According to the European Society for Medical Oncology (ESMO) guidelines (17), Skin toxicity, gastrointestinal toxicity, liver toxicity, pneumonia, and endocrine disruption are common side effects of PD-1 inhibitors. Rare immune-related toxicities include cardiotoxicity, neurotoxicity, rheumatic immunotoxicity, nephrotoxicity, and ophthalmology have also been reported before (18–20). Although the incidence is less than 1%, they often develop in an explosive way, which endangers the life of patients (21) and requires great attention. In a retrospective analysis of a World Health Organization (WHO) pharmacovigilance database (Vigilyze) (4), 52 of 131 fatal immunotherapy-related adverse effects (39.7%) were attributed to myocarditis, and more than a quarter of the fatal myocarditis patients were associated with myositis and MG. PD-1 inhibitor associated myositis/myocarditis with MG often occurs early in treatment and deteriorates rapidly.

Twenty days after tislelizumab therapy, our patient developed weakness of limbs and drooping eyelids, accompanied by chest tightness and pigmented urine. Blood biochemical examination showed that myocardial enzyme, CK and aminotransferase were significantly increased, and CK was more than 50 times the upper limit of normal value. The diagnosis of myasthenia gravis was confirmed because the patient had head drop, facial weakness, ptosis, dysphonia, and shortness of breath, and his anti-AChR antibody was positive. A recent study summarized the onset of ICI-induced MG ranged from 6 to 106 days after the first dose (22). Although patients with Guillain–Barre’ syndrome (GBS) may also present with oculomotor paralysis, limb weakness, and loss of tendon reflex, and GBS has been reported in patients with Non-Hodgkin’s Lymphoma of the colon during chemotherapy (23), GBS in our patient can be excluded as autoimmune peripheral polyneuropathy antibody were negative and EMG was normal. Myasthenia and myositis caused by paraneoplastic syndrome have also been reported (24, 25); however, paraneoplastic antibodies were all negative; therefore, paraneoplastic syndrome can be excluded. Myositis caused by the combination of atorvastatin and nivolumab has also been reported (26); the patient did not take any statins or other drugs that might cause rhabdomyolysis. After comprehensive analysis, ICI-related adverse effects affecting multiple organ systems were considered. Only two cases of tislelizumab-induced irAEs occurred during treatment of small-cell lung cancer and ureteral epithelium (27) have been previously reported (28). Autoantibodies associated with myositis, peripheral polyneuropathy or paraneoplastic syndrome was not detected in both of them. As ICI is rarely used in the treatment of colon cancer, our report provides a new reference for ICI irAEs.

The mechanism of irAEs has not been fully understood to date. It was reported that tumor tissues and striated muscle (myocardium and skeletal muscle) had cross-reactive antigens (29, 30). Consequently, the distinct T-cell receptors misled the immunological system by targeting dissimilar antigens with clonal T-cell receptors across tumor and muscle samples. According to cases of ICI-induced myositis or myocarditis in previous reports, the skeletal muscle and myocardium biopsy revealed a greater number of mononuclear cells, particularly CD8^+^ T cells, resulting in the development of irAEs (22, 29, 31). Cytokines or chemokines released from immune cells can also cause immune-mediated tissue damage (32–35). Researchers have demonstrated that genetic factors play a pivotal role in the development of ICI-associated irAEs in patients with arthritis (36), diabetes (37–39), and pruritus (40). The gut microbiome has been suggested to contribute to experimental irAEs (41, 42) and colitis in melanoma patients (43–45).

MG is a rare antibody-mediated neuromuscular disease, whereby predominant anti-AChR antibodies attack the muscle endplate, leading to fatigable weakness in skeletal muscles (46). Furthermore, in retrospective case-control study researchers found neuromuscular decompensation to be more pronounced in patients with anti–PD-1 treatment (47). It has been more previously reported that patients with a history of MG or positive anti-AChR antibody presented a myasthenic crisis after ICI treatment (48–51) compared with patients without a history of MG. For patients with a history of MG, the use of ICIs might activate the T-cell autoimmune response and may induce MG. For acetylcholine receptor antibody-positive MG patients, high doses of corticosteroids alone can exasperate the myasthenic crisis, and ASCO guideline recommend high doses of corticosteroids combined with gamma globulin or plasma replacement as first-line treatment (17).

IrAEs mainly involves skin, endocrine, liver, gastrointestinal and lung, and other rare immune-related toxicities include nervous system, heart, rheumatoid immunity, and kidney (52). ICI-induced adverse effects are summarized in **Table 1**. Most irAEs, despite being severe in some cases, can be managed and reverted by ceasing immunotherapy and taking steroids, so early diagnosis and treatment is very important (71). Additionally, if a significant irAE is suspected, high-dose corticosteroids must be administered promptly, and patients with persistent symptoms may require escalation to other immunosuppressive therapies. For example, IVIG or plasmapheresis can be used in severe neurologic toxicity. Myocarditis can be treated with infliximab, mycophenolate mofetil, or anti-thymocyte globulin (72–74). Despite the presence of abnormal cardiac biomarker testing, our patient’s surface ECG showed no evidence of atrioventricular conduction delay, and the electrophysiology study showed normal conduction. According to the ASCO guideline (55), these changes were classified as grade III cardiovascular toxicities, suggesting early (i.e., within 24h) initiation of high-dose corticosteroids. Our patient responded well to our treatment. Palpitations and shortness of breath improved, and his troponin gradually returns to normal, so he did not receive additional immunosuppressive therapy. Whenever high-dose corticosteroids fail to produce an immediate response, early institution of cardiac transplant rejection doses of corticosteroids (methylprednisolone 1 g every day) and the addition of either mycophenolate, infliximab, or antithymocyte globulin should be considered (55). Research has shown that the recurrence rate of any grade of irAE is between 25 and 50%. In general, it is recommended that patients suffering from severe irAEs discontinue their ICI treatment permanently (75–77).

**Table 1:** ICI-induced adverse effects, clinical manifestations, and treatment.

Adverse reactions	Clinical manifestations	Treatment
Hepatobiliary diseases (53)	Increased levels of AST, ALT, γ-glutamyl transferase, and bilirubin; autoimmune hepatitis	Hepatic irAEs can require discontinuation of checkpoint inhibitor therapy and treatment with immunosuppressive agents (54).
Endocrine diseases (53, 55)	Hypothyroidism, hyperthyroidism, or other thyroid disorder; adrenocortical insufficiency, diabetes, and secondary hyperglycemia	Some hormone deficiencies can be managed with the corresponding hormone replacement (56).
Blood and lymphatic system disorders (53)	Anemia, leukopenia, neutropenia, thrombocytopenia, hemophilia, and hemolytic anemia	Glucocorticoids are the first line of therapy; IVIG or rituximab can be considered in difficult cases. Neutropenic patients can be treated with G-CSF (57).
Neurological system disorders (19, 58)	Neuromuscular dysfunction (myasthenia gravis), encephalitis, myelitis, cerebral vasculitis, Guillain–Barre Syndrome, and non-infectious meningitis	Corticosteroids were the most frequent treatment, followed by IVIg and plasma exchange (PEX).
Cardiovascular diseases (59)	Pericarditis, myocarditis, and vasculitis	Treatment of ICI-associated myocarditis includes ICI discontinuation, supportive management, and glucocorticoids (60). Prednisone (0.5–2.0 mg/kg), followed by 4–6 weeks taper upon symptoms improvement, is recommended (30, 61).
Respiratory diseases (62, 63)	Pulmonary infections, cough, chest pain, hemoptysis, dyspnea, organizing pneumonia, autoimmune alveolitis, ARDS, and sarcoid-like granulomatosis	Intravenous steroid therapy with intravenous methylprednisolone along with empirical antibiotic therapy should be administered. Bronchoscopy and/or bronchoalveolar lavage are typically performed, and transbronchial biopsy can be considered in a serious condition.
Gastrointestinal diseases (64)	Gastritis, nausea, decreased appetite, esophagitis, gastritis, ileitis, colitis, and pancreatitis	Symptomatic treatment. There is also evidence that infliximab and vedolizumab can be used to treat ICI-induced colitis (35, 65, 66).
Mucosal or cutaneous disease (64)	Rash, pruritus, and vitiligo	The use of topical glucocorticoids is effective for treating low-grade skin reactions, but systemic glucocorticoids are required for high-grade reactions (35).
Musculoskeletal and connective tissue disorders (67, 68)	Arthralgias and rhabdomyolysis	Most patients can be managed with non- steroidal anti-inflammatory drugs or intra-articular glucocorticoid injections (35).
Kidney and urologic diseases (18)	Urinary tract infection and nephritis	Nephrology symptomatic treatment (69).
Ocular diseases (20)	Conjunctivitis, scleritis, episcleritis, uveitis, blepharitis, retinitis, and optic neuritis	Ophthalmic symptomatic treatment (70).
Systemic symptoms (64)	Fever, weight gain, and fatigue	Symptomatic treatment.

Neurological irAEs have unique presentation, including disorders of the central nervous system, peripheral nerves, neuromuscular junctions, and muscles. It is possible for a single patient to have multiple neurological disorders during ICI treatment (78). Myasthenia and myositis are the most common overlap syndromes (79–81). A mixture of MG and myositis can present both clinical manifestations and laboratory findings, such as fatigue, appetite loss, proximal limb weakness, dropped head, dysphagia, respiratory insufficiency, and anti-striational antibodies. As an overlapping condition, myocarditis is also observed (82). In cases where myasthenia and myositis overlap, anti-striational antibodies including titin, ryanodine receptor, muscular voltage-gated potassium channel, Kv1.4 were detected in approximately 75% patients (22, 79, 83). A positive anti-striational antibody rate of 75% is expected to be a biomarker that can be used to diagnose overlapping myasthenia and myositis (84, 85). The anti-titin antibody was positive in our patient, which nicely supported this viewpoint. The T cell–mediated autoimmune mechanism against molecules in the skeletal and heart muscles may be important in the pathogenesis of these overlapping conditions (82). It is possible that peripheral blood may contain T cells that are autoreactive to muscle autoantigens such as titin, Kv1.4, and others (79). ICI treatment activates autoreactive CD8+ T cells, resulting in myositis and myocarditis. Also, autoreactive CD4+ T cells produce anti-AChR and anti-striational antibodies as a result of activation (82).

There is no tendency for neurological irAEs to appear with certain types of underlying cancer, according to epidemiological studies. Neurological irAEs are not associated with the brand of ICI. Nevertheless, atezolizumab can cause autoimmune encephalitis in Asian cancer patients at an unexpectedly high rate (86, 87).

There are several limitations of this case report. First, to further understand the underlying mechanisms of the irAEs, we intended to collect the muscle biopsy specimens from our patient and monitored the cytokine activation and immune cell infiltration. Considering that the muscle biopsy is an invasive test, the patient refused further diagnostic work-up. Second, pulmonary function tests are useful to assess severity of myasthenia gravis at the time of diagnosis and to monitor disease course. It is a pity that our patient cannot cooperate with the pulmonary function test.

Our case report provides experiences in managing multiple irAEs induced by tislelizumab in a patient with colon cancer. Despite its exciting therapeutic prospects, it is becoming an important safety concern. Monitoring immune parameters, making early differential diagnoses, and glucocorticoid therapy as soon as necessary play a crucial role in the treatment of patients who suffer from multiple irAEs, especially when they develop potential lethal complications such as myocarditis.

## Data availability statement

The original contributions presented in the study are included in the article/supplementary material. Further inquiries can be directed to the corresponding author.

## Ethics statement

Written informed consent was obtained from the individual(s) for the publication of any potentially identifiable images or data included in this article.

## Author contributions

Every author has made substantial contributions to the manuscript. SW drafted the article and contributed to editing and revision. DP, HZ, MX, LP, RW contributed to patient follow-up. MZ has substantively edited the manuscript. All authors contributed to the article and approved the submitted version.
